# Organisational change and employee mental health: a conceptual framework integrating conservation of resources theory and the job demands-resources model

**DOI:** 10.3389/fpubh.2026.1925071

**Published:** 2026-09-09

**Authors:** Mmapitsi Louisa Mokwele, Annelize Van Niekerk

**Affiliations:** Department of Industrial and Organisational Psychology, College of Economics and Management Sciences, University of South Africa, Pretoria, Gauteng, South Africa

**Keywords:** communication, employee wellbeing, job demands, job resource, organisational change, psychological safety

## Abstract

Organisational change has intensified in recent years in response to shifting workplace expectations, rapid technological developments, the effects of COVID-19, and evolving work patterns. While such transformation is often necessary for organisational sustainability, it can increase uncertainty, alter job demands, and place emotional pressure on employees. Drawing on Conservation of Resources theory, this article suggests that employees may view change as a threat to valued personal and work resources, heightening stress responses. The Job Demands-Resources model further illustrates how increased demands such as digital overload and rising performance pressures can affect wellbeing unless balanced with appropriate resources, including autonomy, clarity, and supportive work relationships. A range of organisational factors can shape how employees experience these changes. Leadership behaviours, communication practises, opportunities for involvement, and levels of engagement all influence how employees interpret and respond to shifting demands. These factors interact with job demands and resources by shaping perceptions of fairness, predictability, psychological safety, and support during periods of transition. Rather than functioning independently, these elements combine to influence employee wellbeing and adjustment. The article presents a framework in which the impact of organisational change on wellbeing is understood through the interplay between demands, available resources, and the broader relational and communicative environment. It highlights the enduring effects of recent disruptions, which have increased the need to understand how employees adapt to continuous change. The paper concludes by identifying future research directions aimed at improving organisational practises that support employee wellbeing throughout ongoing transformation.

## 1 Introduction

Organisational change has become a defining characteristic of contemporary workplaces, driven by technological advancements, globalisation, and evolving work arrangements. The COVID-19 pandemic has significantly accelerated these changes, transformed organisational structures, and placed psychological pressures on employees (1). Employees are increasingly required to navigate ongoing uncertainty, digital intensification, and shifting expectations, all of which contribute to heightened job demands and mental strain. This intensification of work demands has resulted in increased psychological strain, making employee wellbeing a crucial organisational priority rather than a secondary consideration. The post-pandemic landscape has further exacerbated psychosocial risks, including stress, burnout, and emotional exhaustion. Consequently, employee wellbeing, particularly mental health, has emerged as a pivotal concern for organisations. Despite a growing body of research, there has been limited focus on how post-pandemic organisational change, job demands, and organisational factors interact to affect employee wellbeing. This paper seeks to fill this gap by proposing a conceptual framework grounded in Conservation of Resources (COR) theory and the Job Demands–Resources (JD-R) model.

Although organisational change, employee wellbeing, and workplace mental health have each received considerable scholarly attention, existing research remains fragmented in three important respects. First, studies have generally applied the Conservation of Resources (COR) theory and the Job Demands-Resources (JD-R) model independently, with limited integration of these complementary perspectives in the context of organisational change. Second, while employee wellbeing is frequently examined as a broad organisational outcome, mental health is often treated as a secondary consequence rather than a central dimension of wellbeing. Third, organisational factors such as leadership, communication, employee involvement, and psychological safety have typically been examined in isolation, rather than as interconnected contextual mechanisms that shape how employees experience change-related demands and resources. Consequently, there remains a need for an integrative conceptual framework that explains how organisational change influences employee wellbeing through the dynamic interaction of resource loss, job demands, job resources, and organisational context, particularly within the evolving post-pandemic workplace (2, 3).

Accordingly, the purpose of this paper is to develop a conceptual framework that integrates COR theory and the JD-R model to explain how organisational change influences employee wellbeing and mental health. The framework further proposes that leadership, communication, employee involvement, and psychological safety function as contextual moderators that shape employee responses to changing work conditions.

## 2 Theoretical background

The relationship between organisational change and employee wellbeing can be understood through a theoretical framework that explains how employees perceive and respond to changing work environments. This study draws on both the Conservation of Resources (COR) theory and the Job Demands-Resources (JD-R) model. These frameworks offer complementary perspectives on stress, adaptation, and wellbeing in organisational contexts (4, 5). Together, these theories provide a comprehensive lens through which the organisational and psychological implications of change can be studied.

### 2.1 Conservation of resources (COR) theory

Proposed by Hobfoll (4), COR theory suggests that individuals are driven to obtain, retain, and protect valued resources. These resources include tangible goods such as employment and income, personal resources such as resilience and self-esteem, and social resources such as control and social support. This theory posits that the primary source of stress, particularly in unpredictable and unstable environments, is the loss of resources or fear of such loss. These resources are directly related to the roles, relationships, and working conditions of employees. These resources can be disrupted or threatened by organisational change, creating a perception of instability and uncertainty. For instance, organisational changes such as restructuring, technological transformation, or strategic shifts may create ambiguity regarding job security and role clarity amongst employees. These factors are significantly associated with increased psychological strain and emotional exhaustion (6).

Furthermore, COR theory emphasises that resource loss has greater impact than resource gain, making employees particularly sensitive to loss during periods of change. This is particularly in contemporary work environments are constantly changing and may experience cumulative resource depletion over time. Employees with limited access to resources are therefore more prone to stress and less able to cope effectively with change-related demands (7). In addition, the theory suggests that individuals who utilise their existing resources or acquire new ones, can manage stress by preventing further losses. Increased cognitive and emotional effort is often required during periods of change as employees adapt to new systems and standards. The lack or absence of adequate organisational support, as well as continued resource investment, may lead to burnout and decreased wellbeing.

COR theory and the JD-R model were selected because both place resources at the centre of employee adaptation and wellbeing. Organisational change frequently disrupts access to valued resources while simultaneously increasing workplace demands. Consequently, these frameworks provide complementary perspectives for understanding the psychological and organisational mechanisms through which organisational change influences employee wellbeing (8).

### 2.2 Job demands-resources (JD-R) model

The JD-R model provides a complementary framework for understanding how work-related factors that affect employee wellbeing. This model makes a distinction between job demands and job resources, both of which are crucial in shaping employee outcomes (5, 9). Job demands refer to aspects of work that require sustained physical and psychological effort, such workload, time constraints, role ambiguity, and emotional labour (5, 9). These demands typically increase during periods of change, as employees have to adapt to their new roles, learn new systems, and meet evolving expectations. Prolonged exposure to these increased demands is associated with stress, exhaustion, and reduced wellbeing amongst employees (6, 10).

Job resources, on the other hand, refer to elements of the work environment that assist employees in managing demands, accomplishing tasks, and fostering personal growth (9). These include factors such as autonomy, supportive leadership, information availability, and positive workplace relationships. Job resources function as critical buffering mechanisms that mitigate the adverse effects of job demands while simultaneously promoting engagement and adaptive capacity (7). A key strength of the JD-R model is its ability to explain both negative and positive outcomes through two distinct processes. The motivational process emphasises how job resources enhance engagement and wellbeing, while the wellbeing impairment process illustrates how excessive job demands lead to stress and burnout (9). This dual perspective is particularly useful in comprehending the differences in how employees respond to organisational change.

### 2.3 COR and JD-R integration

Despite originating from different theoretical backgrounds, the COR theory and the JD-R model are closely related and reinforce one another. Both emphasise the crucial role of resources in shaping employee experiences and stress how crucial it is to balance between demands and adequate support. In the context of organisational change, these frameworks can be integrated to explain how organisational change processes increase job demands while also threatening or depleting valued resources. For instance, employees may experience increased workload complexity while losing access to familiar routines, social support, or role clarity. This dual impact creates a demand-resource imbalance that significantly influences employee wellbeing outcomes (7, 10).

The integration of COR theory and the JD-R model offers important conceptual advantages beyond the application of either framework independently. COR theory explains the underlying psychological processes through which organisational change affects employees by emphasising actual or anticipated resource loss. During periods of transformation, employees may perceive threats to valued resources such as job security, role clarity, social support, professional identity, and control over work. These perceived or actual resource losses contribute to stress, uncertainty, and reduced wellbeing.

While COR theory provides a valuable explanation of why organisational change may be experienced as psychologically demanding, it provides less detail regarding the specific work characteristics that influence employee outcomes. The JD-R model complements this perspective by identifying the mechanisms through which job demands and job resources influence wellbeing. Within the JD-R framework, organisational change may increase job demands through workload intensification, role ambiguity, cognitive complexity, and emotional strain, while job resources such as autonomy, information, and social support may help employees adapt to these demands.

Integrating these perspectives provides a more comprehensive explanation of employee wellbeing during organisational change. COR theory explains the psychological significance of resource loss and resource protection, whereas the JD-R model explains how job demands and job resources shape employee outcomes within changing work environments. Together, these frameworks offer a stronger theoretical foundation for understanding how organisational change influences employee wellbeing and mental health through the interaction of resource dynamics, workplace demands, and organisational support mechanisms.

Integrating these perspectives offers a more nuanced understanding of the structural and psychological processes underlying employees' reactions to organisational change. Importantly, this integrated approach emphasises that negative outcomes are not inevitable. The impact of increased demands can be mitigated, and positive adaptation can be supported through access to adequate resources, such as opportunities for involvement, clear communication, and leadership support. Accordingly, the proposed conceptual framework draws on COR theory to explain the psychological consequences of threatened or depleted resources, while the JD-R model provides the structural basis for understanding how demands and resources interact to influence employee wellbeing during organisational change. The complementary contributions of Conservation of Resources (COR) theory and the Job Demands-Resources (JD-R) model are summarised in Table 1.

**Table 1:** Complementary contributions of COR and JD-R.

COR theory	JD-R model
Explains why change causes stress	Explains how stress develops
Focuses on resource loss	Focuses on demand-resource balance
Psychological mechanism	Work design mechanism
Emphasises resource protection	Emphasises buffering resources

### 2.4 The role of organisational context

Both COR theory and the JD-R model acknowledge that employee experiences are influenced by both by individual factors and by the broader organisational context. Organisational culture, leadership style, communication practises, and workplace relationships all affect how employees perceive and respond to change. For instance, clear and consistent communication improves employees' sense of control, thus reducing uncertainty (24). Similarly, supportive leadership serves as an essential organisational resource by offering both emotional reassurance and practical guidance (11). Furthermore, during periods of change, trust and wellbeing are significantly shaped by organisational justice. Organisational justice refers to employees' perceptions of fairness (12).

The American Psychological Association (13) further highlights one other element that plays a crucial role during organisation transformation, namely, psychological safety. Psychological safety refers to a shared belief that the workplace is safe for interpersonal risk-taking, allowing employees to speak up without fear of negative consequences (20, 25). When employees feel safe to voice concerns and engage openly without fear of negative consequences, they are better equipped to adjust and manage stress (14). Conversely, low psychological safety can exacerbate uncertainty and emotional strain. These organisational factors serve as contextual resources, influencing the perception of how stressful or manageable job demands are.

### 2.5 Linking theory to the conceptual framework

This paper conceptualises organisational change as a process that disrupts the balance between job demands and resources. Drawing on COR theory and the JD-R model, these theories suggest that increased job demands, and perceived loss of resources exacerbate stress and reduced wellbeing amongst employees, whereas the availability of resources can buffer these negative effects and facilitate adaptation (6). This occurs because employees experiencing heightened demands must expend greater effort to cope, which depletes their psychological and emotional resources, thereby increasing vulnerability to adverse outcomes. In this context, employee wellbeing is conceptualised as a multifaceted concept with mental health as a crucial component. Consequently, outcomes such as stress, burnout, and emotional exhaustion reflect the psychological cost of organisational change, particularly when demands are higher than available resources (6). Therefore, this integrated theoretical perspective provides the foundation for the conceptual framework presented in this study, emphasising the dynamic interaction between organisational change, job demands, job resources, and organisational context.

## 3 Organisational change and employee wellbeing

Organisational change can be defined as a process through which organisations transition from its current form to a desired future state. These changes may involve structural, technological, cultural, or strategic transformations with the goal of improving the sustainability and performance of the organisation. While these changes are essential in increasingly dynamic workplaces, they often introduce significant challenges for employees. For example, organisational change initiatives such as digital transformation and technology-driven restructuring may increase employee job demands and add complicity change processes (2, 5, 24).

From an employee perspective, organisational change is rarely experienced as natural or purely operational process. Rather, it is often perceived as disruptive, demanding employees to manage constant uncertainty while adjusting to new roles, processes, and expectations. These demands have become increasingly apparent in post-pandemic work environments, where continuous and cumulative changes have increased employees' exposure to uncertainty and pressure (6).

Employee wellbeing is widely recognised as a multidimensional construct encompassing psychological, emotional, and social functioning. It encompasses both positive experiences such as fulfilment and engagement, as well as negative experiences such as stress, dissatisfaction, and burnout. According to Zeshan et al., (10), wellbeing has a significant impact on commitment, performance, productivity, commitment, and turnover intentions.

The relationship between organisational change and employee wellbeing is therefore complex and multifaceted (15). Change processes often result in increased job demands, such as heightened performance expectations, role ambiguity, and workload intensification. These demands in turn necessitate sustained cognitive and emotional effort, which may be more than employees' coping capacity, especially when resources are limited (7, 10). Moreover, organisational change may cause a disruption towards employees' sense of security and predictability, which further exacerbates psychological strain and emotional exhaustion (6).

### 3.1 The mental health as a core dimension of employee wellbeing

Mental health represents a crucial and increasingly prominent dimension of the broader concept of employee wellbeing. It can be understood as employees' psychological and emotional functioning, encompassing both positive states such as resilience and engagement, as well as negative outcomes such as stress, anxiety, and burnout (16, 17). Organisational change has consistently been associated with adverse mental health outcomes, especially when employees are exposed to prolonged periods of uncertainty and increased demands (1). Changes such as restructuring or technological transformation often create perceptions of job insecurity and reduced control, which are key drivers of psychological distress (6, 18).

For conceptual clarity, employee wellbeing is defined in this study as a multidimensional construct encompassing psychological, emotional, and social functioning (10, 19). Mental health represents a central dimension of employee wellbeing and reflects employees' psychological capacity to function effectively and cope with work-related demands (3, 16). Within this framework, outcomes such as stress, anxiety, emotional exhaustion, and burnout are not treated as synonymous with wellbeing but rather as indicators of impaired mental health and reduced wellbeing. Stress refers to psychological strain arising from perceived demands that exceed available coping resources. Emotional exhaustion reflects feelings of emotional depletion resulting from prolonged exposure to work-related demands, whereas burnout represents a broader work-related syndrome characterised by emotional exhaustion, cynicism, and reduced professional efficacy. Distinguishing between these constructs improves conceptual precision and provides a clearer basis for understanding how organisational change influences employee wellbeing and mental health within the proposed framework.

These stressors become more severe when employees lack adequate resources or support to effectively manage change. The challenges have intensified in the post-pandemic environment, where employees are required to rapidly adapt to evolving work conditions, often without sufficient time or support, resulting in cumulative psychological strain (2, 17). Over time, this prolonged exposure to high demands and limited resources may lead to emotional exhaustion and burnout, characterised by depletion, detachment, and reduced professional efficacy (7, 20, 21).

It is important to recognise that mental health outcomes are not determined solely by the presence of stressors, but also by the availability of organisational resources and support systems. Mechanisms such as social support and perceptions of organisational justice play a critical role in mitigating stress and promoting psychological wellbeing (12, 15). Employees who perceive higher levels of support and fairness are more likely to report improved mental health outcomes and greater resilience during organisational change (12). Therefore, mental health should be understood as an integral component of employee wellbeing, shaped by the interaction between job demands and organisational conditions, rather than as a secondary outcome (2).

### 3.2 The role of demand-resource imbalance in wellbeing outcomes

The impact of organisational change on employee wellbeing can further be understood through the disequilibrium between job demands and available job resources. Demands often increase rapidly during periods of change, while resources either remain stable or decline. This imbalance reflects both a depletion of valued resources and an excess of job demands relative to available support, thereby intensifying vulnerability to stress and burnout (5, 10, 20). This dynamic is particularly significant because it highlights not only the level of demands placed on employees but also their capacity to cope with those demands. Consequently, when employees lack access to resources such as information, managerial support, or autonomy, their ability to adapt is compromised, increasing the likelihood of stress, disengagement, and dissatisfaction (7).

Conversely, when organisations provide adequate resources, the negative effects of increased demands can be mitigated. For example, clear communication enhances predictability, employee involvement strengthens perceived control, and supportive leadership provides both emotional and practical support (11, 20). These resources enable employees to interpret change-related demands as manageable challenges rather than overwhelming stressors. The interaction between demands and resources is therefore central to understanding employee wellbeing during organisational change. Rather, it highlights that the results of change are not predetermined, but they are shaped by how well job demands and available resources are well balanced.

## 4 Organisational factors influencing change

Organisational change introduces structural and job-related challenges; however, the organisational context in which these changes occur significantly shapes how employees perceive these changes. Organisational factors play a central role in influencing how employees interpret, respond to, and cope with change. For example, these factors determine whether change is viewed as a manageable transition or as a significant source of stress and disruption (6). This highlights that organisational change is not purely technical or procedural, but rather a socially constructed process shaped by interactions between employees, leaders, and organisational systems. Consequently, factors such as leadership behaviour, communication practises, and employee involvement are particularly influential in shaping employee wellbeing during periods of transformation (10).

### 4.1 Leadership and employee experience during change

Leadership plays a pivotal role in shaping employee experiences of organisational change. Beyond implementing change initiatives, leaders are responsible for guiding employees through the transition process. As such, leadership behaviours can either mitigate or intensify the psychological impact of change. Supportive leadership is especially important during periods of uncertainty (11). Leaders who provide clear direction, demonstrate empathy, and actively support employees contribute to a sense of stability and reassurance. These behaviours function as key organisational resources, enabling employees to cope with increased demands and reducing the likelihood of negative wellbeing outcomes (11).

In contrast, ineffective leadership, characterised by inconsistent communication, lack of support, or limited transparency can exacerbate uncertainty and contribute to negative employee experiences. Under such circumstances, employees may feel connected and less confident in their ability to adapt, which can negatively affect both wellbeing and engagement (6). Furthermore, leadership also influences employees' perceptions of fairness and organisational support. When leaders are perceived as transparent and inclusive, employees are more likely to trust organisational processes and engage positively with change initiatives. This highlights the crucial role of leadership as both a direct and indirect determinant of employee wellbeing during change.

### 4.2 Communication and the reduction of uncertainty

Communication is a critical organisational factor influencing employee responses to change. Specifically, organisational change is often accompanied by uncertainty regarding roles, expectations, and organisational direction (20). In such circumstances, when clear communication is absent, employees may rely on assumptions or informal information sources, which can increase anxiety and confusion (20, 24). Consequently, effective communication becomes essential, as it reduces ambiguity and enhances predictability by providing employees with clear and timely information about change processes (15, 20). In doing so, it reduces role ambiguity and uncertainty, functioning as a key psychosocial resource that supports employee coping during organisational change. This, in turn, improves employees' sense of control and directly contributes to reduced psychological strain (6). Given that uncertainty is a key driver of stress, communication therefore plays a central role in protecting employee mental health during organisational change.

Furthermore, the effectiveness of communication extends beyond the content delivered to include the way it is conveyed. Two-way communication fosters trust and inclusion by enabling employee voice, which is central to the development of psychologically safe and adaptive work environments. Transparent, open, and two-way communication allows employees to ask questions, express concerns, and seek clarification (15, 20). As a result, this participatory approach strengthens psychological safety and inclusion (13). Ultimately, such communication practises reinforce employees' ability to cope with change, thereby supporting both engagement and wellbeing.

### 4.3 Employee involvement and participation

Employee involvement is another key organisational factor that influences wellbeing during change. This involves allowing employees to participate in decision-making processes, allowing them to have a voice in shaping change initiatives. By so doing, employees are more likely to develop an increased sense of ownership and control over organisational changes, which in turn enhances their commitment and reduces resistance to change. Furthermore, when employees are actively involved in change processes, they are more likely to perceive change as fair and legitimate. This perception of fairness, often conceptualised as organisational justice, has been shown to significantly influence employee wellbeing and engagement (12). Consequently, employees who perceive higher levels of organisational justice are more likely to experience positive mental health outcomes and demonstrate resilience during organisational change.

Contrary to the above, limited involvement can negatively affect employee wellbeing during the change. When employees feel excluded from the decision-making processes, they may feel powerless, experience heightened uncertainty, and reduced trust. In turn, these experiences may lead to employee disengagement and negatively affect their wellbeing, as employees may feel coerced towards the change. Importantly, involvement does not necessarily require full decision-making authority. Even opportunities for consultation, feedback, and participation in implementation processes can serve as valuable resources that support employee adaptation and psychological wellbeing. Therefore, ensuring even moderate levels of employee involvement is essential for mitigating the negative psychological effects of organisational change and supporting employee wellbeing.

### 4.4 Psychological safety and organisational support

Psychological safety is a critical organisational factor during periods of change, as employees are required to navigate uncertainty, new processes, and evolving expectations. When employees feel psychologically safe, they are more likely to engage in open communication and active problem-solving, which enhances their capacity to adapt to change and manage stress (13, 14). Conversely, low levels of psychological safety can heighten anxiety and hinder engagement, ultimately undermining employee wellbeing. Psychological safety refers to a shared belief that the workplace is safe for interpersonal risk-taking, allowing individuals to express themselves without fear of negative consequences such as embarrassment or punishment (20, 25). In this context, it enables employees to engage in behaviours such as voicing concerns and seeking clarification, which are essential for effective adaptation in high-change environments.

Furthermore, organisational support mechanisms play a vital role in reinforcing psychological safety during organisational change. These may include formal interventions such as employee assistance programmes, digital wellbeing tools, and organisational policies designed to support mental health. Such initiatives signal organisational commitment to employee wellbeing and help build trust, particularly in contexts where technological and structural changes increase uncertainty (15).

### 4.5 Integration of organisational factors

The organisational factors discussed above (leadership, communication, employee involvement, and psychological safety) can be conceptualised as critical resources within the JD-R framework and as key mechanisms influencing resource availability in COR theory. As contextual resources, these factors shape how employees interpret and respond to job demands, ultimately determining whether organisational change is experienced as manageable or overwhelming. They interact dynamically with job demands and individual resources to influence employee wellbeing outcomes (15, 20).

For example, effective leadership can reduce perceived demands while enhancing access to resources (11); clear communication mitigates uncertainty and improves role clarity (24); employee involvement increases perceived control and reduces stress (12); and psychological safety buffers emotional strain while supporting adaptation (13). Through these mechanisms, organisational factors act as important moderators in the relationship between job demands and employee wellbeing. They influence how employees appraise change-related demands and whether these demands lead to stress and disengagement or are effectively managed through available support (10).

### 4.6 Implications for understanding employee wellbeing in the context of organisational change

The inclusion of organisational factors highlights the need to view organisational change as a holistic process that extends beyond structural and operational considerations. Employee wellbeing during change is shaped by the interaction of job demands, available resources, and the organisational context in which change occurs (6, 7, 12).

This perspective reinforces the importance of adopting a human-centred approach to organisational change, where employee experiences are actively considered and supported. By recognising the role of organisational factors, organisations can better design and implement change initiatives that minimise negative outcomes while promoting employee wellbeing. These insights provide a direct foundation for the conceptual framework presented in the next section, which integrates organisational factors with job demands and resources to explain outcomes associated with employee wellbeing.

## 5 Conceptual framework of organisational change, employee wellbeing, and mental health

The preceding discussion has highlighted how organisational change influences employee wellbeing through resource dynamics, job demands, and organisational context. Synthesising these insights, the next section presents an integrated conceptual framework that explains the relationships among these constructs.

**Figure 1:**
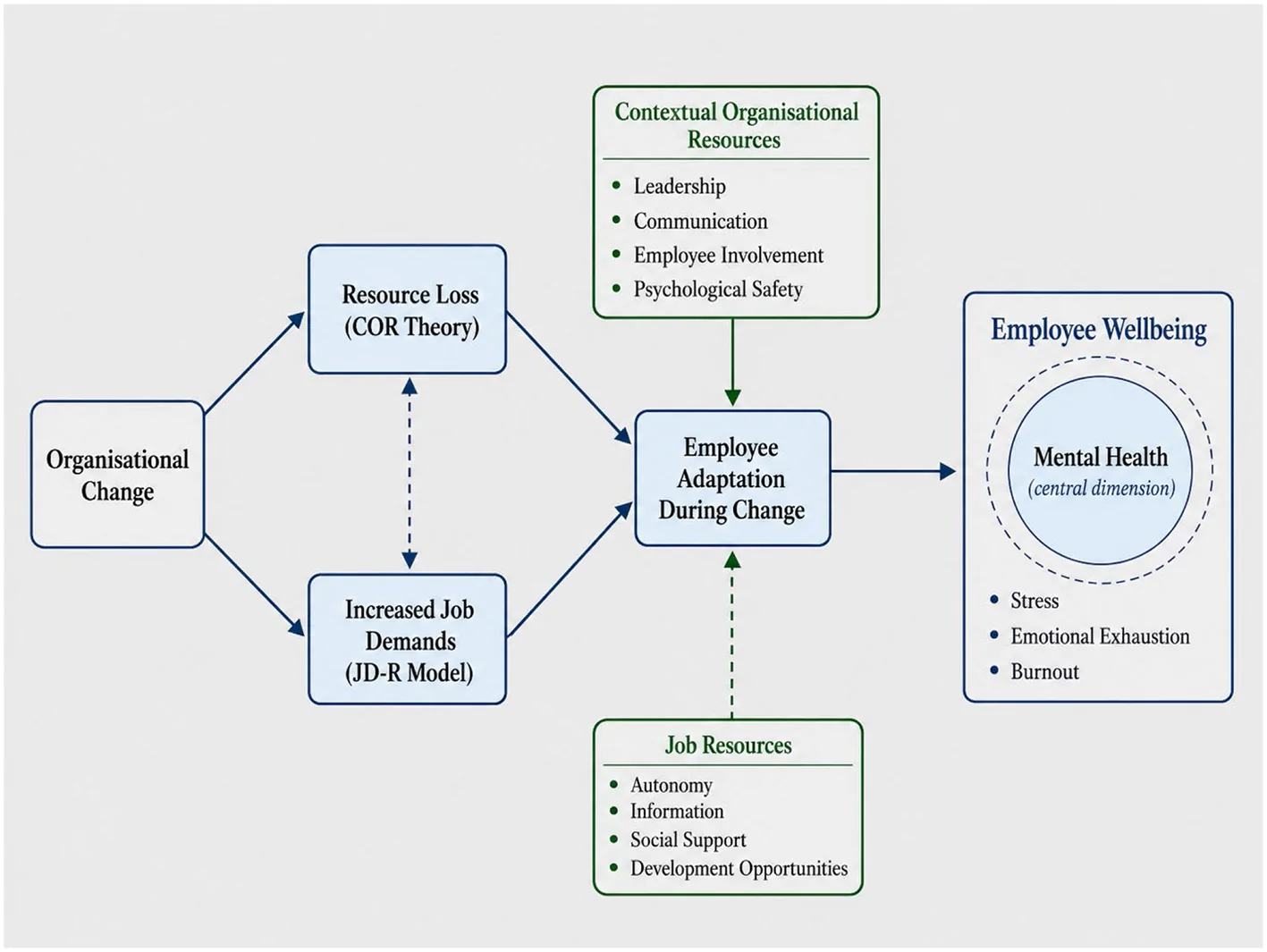
A conceptual framework of organisational change, employee wellbeing, and mental health. Organisational change is proposed to influence employee wellbeing and mental health through the interaction of resource loss (COR theory), increased job demands and job resources (JD-R model), and contextual organisational resources. Employee adaptation during change represents the central process through which employees respond to changing work conditions. Mental health is conceptualised as a central dimension of employee wellbeing, with stress, emotional exhaustion, and burnout representing indicators of impaired wellbeing outcomes.

The proposed conceptual framework illustrating the relationships between organisational change, resource loss, job demands, job resources, employee adaptation, and wellbeing outcomes is presented in Figure 1. Building on the theoretical foundations of Conservation of Resources (COR) theory and the Job Demands-Resources (JD-R) model, as well as the organisational factors discussed in preceding sections, this paper proposes a conceptual framework to explain how organisational change influences employee wellbeing. The framework integrates multiple dimensions of the employee experience, highlighting the dynamic interaction between organisational change, job demands, job resources, and organisational context (4, 5).

At its core, the framework conceptualises organisational change as a primary driver of altered work conditions. Organisational change acts as a structural catalyst that increases job demands while simultaneously destabilising access to established resources, thereby creating conditions that directly influence employee wellbeing outcomes. Organisational change initiatives such as restructuring, technological transformation, and strategic realignment introduce shifts in roles, expectations, and work processes. These shifts increase job demands and disrupt existing resources, thereby creating conditions that influence employee wellbeing and mental health outcomes (6, 24).

To improve conceptual clarity, the framework distinguishes between antecedents, explanatory mechanisms, contextual organisational resources, and outcomes. Organisational change is conceptualised as the primary antecedent that initiates alterations in work conditions. Resource loss and increased job demands represent the principal mechanisms through which organisational change may influence employee wellbeing. Job resources function as buffering resources that enable employees to cope with changing work requirements and mitigate the negative effects of demands. Employee wellbeing, with mental health as a central dimension, represents the primary outcome of the framework.

### 5.1 Organisational change as a driver of job demands

A central proposition of the framework is that organisational change increases job demands. Employees are required to adapt to new systems, acquire new competencies, and respond to evolving performance expectations. These requirements increase both cognitive and emotional demands, placing pressure on employees' capacity to cope (7). In addition to task-related demands, organisational change introduces psychosocial stressors such as uncertainty, reduced role clarity, and perceived instability. These stressors are closely associated with negative mental health outcomes, including anxiety, stress, and burnout (6). From a COR perspective, these conditions represent resource loss or the threat of resource loss, further intensifying psychological strain.

### 5.2 Employee wellbeing as a multidimensional outcome

The framework positions employee wellbeing, with mental health as a central dimension, as the primary outcome of organisational change processes (2, 15, 20). In line with existing literature, wellbeing is understood as a multidimensional construct that includes psychological, emotional, and social functioning (10). Within this framework, mental health is explicitly acknowledged as a critical dimension. Issues such as stress, emotional exhaustion, anxiety, and diminished resilience reflect the psychological toll of organisational change, especially in scenarios where employees face sustained demands coupled with insufficient resources (2, 6). This integrated perspective highlights that mental health should not be viewed as a secondary outcome but as a key indicator of how employees navigate organisational change.

### 5.3 The moderating role of job resources

While organisational change increases job demands, its impact on employee wellbeing is not uniform. The framework emphasises the moderating role of job resources in shaping this relationship. Job resources (including autonomy, access to information, social support, and developmental opportunities) enable employees to cope with increased demands and adapt to changing work environments (5, 7, 9). Job resources moderate the relationship between organisational change and employee wellbeing by buffering strain and facilitating adaptive responses to shifting work demands.

Within the JD-R model, these resources serve two key functions: a buffering function that reduces the negative effects of job demands on stress and burnout, and a motivational function that enhances engagement, resilience, and overall wellbeing (9, 10). During organisational change, the availability of job resources is particularly critical. When sufficient resources are present, employees are more likely to perceive change-related demands as manageable challenges rather than overwhelming stressors.

### 5.4 Organisational factors as contextual moderators

In addition to job resources, the framework incorporates organisational factors as higher-level contextual moderators. These include leadership, communication, employee involvement, and psychological safety.

These factors shape how employees interpret and respond to job demands:

- Leadership influences perceptions of support, guidance, and fairness (11).
- Communication reduces uncertainty and enhances clarity (24).
- Employee involvement increases perceived control and ownership (12).
- Psychological safety enables open engagement and reduces emotional strain (15, 20).

Within the present framework, leadership, communication, employee involvement, and psychological safety are conceptualised as contextual organisational resources. Although these factors may also function as job resources at the individual level, they are treated here as higher-order organisational influences because they shape employees' access to, interpretation of, and response to job demands and job resources during organisational change. Consequently, these factors are proposed to influence the extent to which organisational change is experienced as manageable, challenging, or psychologically stressful. Through these mechanisms, organisational factors determine whether job demands result in negative outcomes, such as stress and disengagement, or are effectively managed through available support systems (10).

### 5.5 Dynamic interaction between framework components

A defining feature of the proposed framework is its emphasis on dynamic interaction rather than linear causality. Organisational change, job demands, job resources, and organisational factors interact continuously to shape employee wellbeing outcomes. For example, similar levels of job demand may produce different outcomes depending on the availability of resources and the organisational context. In supportive environments, employees may experience change as an opportunity for growth. In contrast, in resource-constrained environments, the same changes may lead to stress and burnout (10). This perspective reflects the complexity of organisational change and underscores the importance of considering multiple interacting factors when examining employee wellbeing (10, 22).

### 5.6 Conceptual model overview

The conceptual framework can be summarised as follows:

- Organisational change acts as a primary driver of resource loss and increased job demands.
- Perceived or actual resource loss represents a key psychological mechanism linking organisational change and employee wellbeing.
- Increased job demands influence employee wellbeing and mental health outcomes.
- Job resources function as buffers, mitigating the negative effects of demands.
- Organisational factors act as contextual moderators, shaping employee perceptions and responses.
- Employee wellbeing is conceptualised as a multidimensional construct, with mental health as a central component.

This integrative model provides a comprehensive explanation of how organisational change influences employee wellbeing through the interaction of demands, resources, and organisational context. The key constructs, definitions, theoretical origins, and roles included in the proposed conceptual framework are summarised in Table 2.

**Table 2:** Key constructs included in the proposed conceptual framework.

Construct	Definition	Theoretical origin	Role in framework	Examples/Indicators
Organisational change	Structural, strategic, technological, or cultural changes affecting work environments (6)	Organisational change literature	Antecedent	Restructuring, digital transformation, policy reform
Resource loss	Actual or perceived loss of valued resources (4)	COR theory	Psychological mechanism	Job insecurity, reduced control, loss of support
Job demands	Aspects of work requiring sustained effort (5)	JD-R model	Explanatory mechanism	Workload, role ambiguity, performance pressure
Job resources	Factors that support coping and adaptation (9)	JD-R model	Buffering resource	Autonomy, information, social support
Leadership	Leader behaviours that influence employee experiences of change (11)	Leadership literature	Contextual organisational resource	Guidance, support, transparency
Communication	Information exchange during change processes (20)	Communication literature	Contextual organisational resource	Clarity, transparency, timeliness
Employee involvement	Participation in change-related decisions (12)	Organisational justice literature	Contextual organisational resource	Consultation, feedback opportunities
Psychological safety	Shared belief that employees can speak openly without fear of negative consequences (25)	Psychological safety theory (25)	Contextual organisational resource	Voice, open dialogue, learning behaviours
Employee wellbeing	Psychological, emotional, and social functioning (2, 10)	Wellbeing literature	Primary outcome	Engagement, functioning, resilience
Mental health	Psychological dimension of wellbeing (16, 17)	Occupational health psychology	Outcome domain	Psychological functioning, coping capacity
Stress	Psychological strain resulting from excessive demands (4)	Stress theory	Wellbeing indicator	Perceived strain
Emotional exhaustion	Feelings of emotional depletion (5, 21)	Burnout literature	Wellbeing indicator	Fatigue, depletion
Burnout	Syndrome characterised by exhaustion, cynicism, and reduced efficacy (5)	Jd-r/burnout literature	Wellbeing indicator	Emotional exhaustion, disengagement

### 5.7 Research propositions

As this study presents a conceptual framework rather than an empirical investigation, the relationships described below are proposed based on theoretical reasoning and prior research and should be regarded as propositions requiring future empirical verification.

**Proposition 1:** Organisational change is proposed to increase job demands and threaten access to valued resources, thereby creating conditions that may negatively affect employee wellbeing.

**Proposition 2:** Increased job demands are proposed to be associated with poorer employee wellbeing and mental health outcomes, including heightened stress, emotional exhaustion, and burnout.

**Proposition 3:** Job resources are proposed to buffer the negative effects of job demands by supporting employee adaptation and coping during organisational change.

**Proposition 4:** Leadership, communication, employee involvement, and psychological safety are proposed to function as contextual organisational resources that moderate the relationship between organisational change, job demands, and employee wellbeing.

## 6 Discussion

The purpose of this paper was to develop a conceptual framework that explains how organisational change influences employee wellbeing through the interaction of job demands, job resources, and organisational factors. Drawing on Conservation of Resources (COR) theory and the Job Demands–Resources (JD-R) model, the proposed framework provides an integrated understanding of how employees experience and respond to change in contemporary organisational contexts (4, 5).

The proposed framework advances organisational change scholarship by conceptualising organisational change as both a structural and psychological process. Rather than viewing change solely as an operational activity, the framework highlights how organisational transformation can alter resource availability, increase workplace demands, and influence employee wellbeing through interconnected demand-resource dynamics (6, 22).

Consistent with COR theory, the proposed framework suggests that actual or anticipated resource loss represents a key mechanism through which organisational change influences employee wellbeing (4, 8). Employees may experience disruptions to key resources such as job security, role clarity, and social support, which in turn contribute to psychological strain and emotional exhaustion (7). This reinforces the value of COR theory in explaining why organisational change can have disproportionately negative effects on employee mental health, particularly in environments characterised by instability (7, 10).

Drawing on the JD-R model, the framework further proposes that employee wellbeing during organisational change is influenced by the balance between job demands and available resources. Organisational change often increases cognitive, emotional, and behavioural demands while creating uncertainty about resource availability. The framework suggests that when demands exceed available resources, employees may experience heightened stress, emotional exhaustion, and reduced wellbeing. Conversely, adequate job resources, such as autonomy, information, and social support, may facilitate adaptation and resilience during periods of change (5, 9).

Another significant contribution of the study is the explicit integration of mental health within the broader construct of employee wellbeing. The framework positions mental health outcomes such as stress, anxiety, and emotional exhaustion, not as secondary consequences, but as central indicators of how employees experience organisational change. This perspective is particularly relevant in the post-COVID-19 context, where workplace mental health has emerged as a key organisational priority (2).

A distinguishing feature of the proposed framework is the inclusion of leadership, communication, employee involvement, and psychological safety as contextual organisational resources. Rather than examining these constructs independently, the framework proposes that they collectively shape how employees interpret organisational change, access resources, and respond to changing work demands (11, 23).

The framework also underscores the variability of employee responses to organisational change. Individual experiences are shaped by the interaction of job demands, resources, and organisational context, rather than by change itself. This highlights the importance of adopting context-sensitive approaches to change management that account for diverse employee experiences (6).

Furthermore, the discussion draws attention to the evolving nature of work, particularly in relation to digital transformation and technological intensity. These developments have introduced new forms of job demands, including cognitive complexity, continuous connectivity, and performance monitoring, which have important implications for employee wellbeing (24). As a result, traditional models of work stress must be extended to reflect the realities of digitally mediated work environments.

From a practical perspective, the proposed framework reinforces the importance of adopting a human-centred approach to organisational change. Organisations that prioritise structural outcomes without addressing employee experiences risk undermining both wellbeing and performance. In contrast, organisations that actively provide resources, foster supportive environments, and promote psychological safety are more likely to achieve sustainable change outcomes (2, 12).

Finally, the study highlights the interdependence between employee wellbeing and organisational effectiveness. Supporting employee wellbeing is not only an ethical responsibility but also a strategic imperative, as it directly influences productivity, engagement, and retention. This reinforces the importance of embedding wellbeing considerations into organisational change strategies.

### 6.1 Theoretical contributions and implications

The proposed framework makes four primary theoretical contributions. First, it integrates Conservation of Resources theory and the Job Demands-Resources model within a single explanatory framework of organisational change and employee wellbeing. Second, it positions mental health as a central dimension of employee wellbeing rather than as a secondary outcome. Third, it conceptualises leadership, communication, employee involvement, and psychological safety as contextual organisational resources that shape employee experiences of change. Finally, the framework advances current understanding by proposing a dynamic interaction between organisational change, resource loss, job demands, and organisational context, thereby offering a more comprehensive explanation of employee wellbeing during organisational transformation.

The literature reviewed in this study highlights several important insights regarding organisational change and employee wellbeing. First, organisational change is consistently associated with increased job demands, uncertainty, and psychological strain (6, 7). Second, employees exposed to organisational change may experience actual or perceived resource loss, which contributes to stress and diminished wellbeing (4, 8). Third, job resources such as autonomy, information, and social support can buffer the adverse effects of increased job demands and support employee adaptation (5, 9). Finally, organisational factors including leadership, communication, employee involvement, and psychological safety influence how employees interpret and respond to organisational change (11, 12, 25).

Despite these findings, several important gaps remain within the existing literature. Previous studies have generally applied Conservation of Resources (COR) theory and the Job Demands-Resources (JD-R) model independently, limiting understanding of how resource loss processes and demand-resource dynamics interact during organisational change. Mental health has also frequently been treated as a secondary outcome rather than as a central dimension of employee wellbeing. Furthermore, organisational factors such as leadership, communication, employee involvement, and psychological safety have often been examined in isolation rather than as interconnected organisational resources. Finally, limited conceptual attention has been given to understanding how these factors collectively influence employee adaptation during organisational change within contemporary post-pandemic work environments.

The proposed framework addresses these gaps by integrating COR theory and the JD-R model within a single conceptual structure. It further positions mental health as a central dimension of employee wellbeing and conceptualises leadership, communication, employee involvement, and psychological safety as contextual organisational resources. In doing so, the framework provides a more comprehensive explanation of how organisational change influences employee wellbeing through the interaction of resource loss, job demands, job resources, organisational context, and employee adaptation.

### 6.2 Implications comparison with existing frameworks

Existing frameworks have generally focused either on resource dynamics and resource preservation processes (COR theory) or on the balance between job demands and job resources (JD-R model) (4, 5, 9). While these perspectives have contributed significantly to understanding employee wellbeing, they tend to explain either psychological resource processes or work-design mechanisms in relative isolation. The present framework extends existing approaches by integrating these perspectives and incorporating contextual organisational resources such as leadership, communication, employee involvement, and psychological safety. In doing so, it provides a more holistic understanding of how organisational change influences employee wellbeing and mental health through the interaction of resource loss, job demands, job resources, and organisational context (6, 22, 23).

## 7 Practical implications

The conceptual framework proposed in this paper has several important implications for organisational practise, particularly for leaders, human resource practitioners, and organisational psychologists involved in managing change processes. As employee wellbeing is increasingly recognised as a critical determinant of organisational effectiveness, organisations need to adopt strategies that mitigate the negative effects of change while promoting positive psychological outcomes (10). A central implication of this study is the need for organisations to adopt a proactive and human-centred approach to organisational change. Rather than treating change as a purely structural or technical process, organisations should recognise that employees' psychological experiences play a critical role in determining the success of change initiatives. This requires integrating employee wellbeing considerations into the planning and implementation of change strategies (2).

Organisations should prioritise structured and transparent communication practises as a strategic intervention to reduce uncertainty, enhance psychological safety, and support employee wellbeing during change. This highlights the importance of aligning organisational processes with employee needs, ensuring that change initiatives are supported not only operationally but also psychologically.

### 7.1 Strengthening communication practises

Effective communication is essential for reducing uncertainty and supporting employee wellbeing during organisational change. Organisations should prioritise transparent, timely, and consistent communication throughout all stages of the change process. Providing clear information about the purpose, scope, and expected outcomes of change initiatives enhances employees' sense of predictability and control, thereby reducing psychological strain (6). Beyond information dissemination, organisations should encourage two-way communication, allowing employees to ask questions, express concerns, and provide feedback. This participatory approach fosters trust, reduces ambiguity, and strengthens employees' sense of inclusion, all of which are critical for maintaining wellbeing during periods of transformation (24).

Importantly, communication should not be treated as a once-off activity but as an ongoing process that evolves alongside organisational change. Continuous engagement with employees ensures that concerns are addressed in real time and that employees remain informed, involved, and supported throughout the transition. In this way, communication functions not only as an information-sharing tool but also as a key organisational resource that enables adaptation, resilience, and sustained employee wellbeing.

### 7.2 Enhancing leadership capability

Leadership plays a critical role in shaping employee experiences of organisational change. Organisations should invest in developing leaders who can provide both technical guidance and psychological support. This includes equipping leaders with skills to manage uncertainty, communicate effectively, and demonstrate empathy in their interactions with employees (11). Supportive leadership behaviours such as acknowledging employee concerns, providing clear guidance, and encouraging participation function as key resources that buffer the negative effects of increased job demands. Leaders who demonstrate transparency, fairness, and consistency contribute to a stable and supportive organisational environment, which is essential for promoting employee wellbeing (6).

### 7.3 Promoting employee involvement and participation

Employee involvement is essential for improving both adaptation and wellbeing during organisational change. Organisations should actively involve employees in decision-making processes, particularly where changes directly affect their roles and responsibilities. Participation can take various forms, including consultation, collaborative problem-solving, and involvement in implementation processes. When employees have opportunities to contribute, they are more likely to perceive change as fair and legitimate. This perception of organisational justice has been shown to enhance employee resilience and mental health outcomes (12). Conversely, limited involvement may lead to perceptions of exclusion, reduced trust, and increased psychological strain. Designing inclusive change processes that recognise and value employee contributions is therefore critical for promoting positive outcomes.

### 7.4 Supporting mental health and wellbeing

Given the central role of mental health within employee wellbeing, organisations should implement targeted interventions that support psychological functioning during periods of change. These may include employee assistance programmes, counselling services, stress management initiatives, and organisational wellbeing policies (2). Equally important is the development of a workplace culture that promotes psychological safety, where employees feel comfortable expressing concerns and seeking support without fear of negative consequences (13). Such environments enable open communication, learning, and adaptation, all of which are essential during organisational change. Organisations should also recognise that mental health is shaped not only by individual factors but also by organisational conditions. Addressing structural factors - such as workload, role clarity, and access to resources - is therefore critical for achieving sustainable wellbeing outcomes.

### 7.5 Balancing job demands and resources

A critical implication of the proposed framework is the need to actively manage the balance between job demands and job resources. During organisational change, demands often increase rapidly, creating a risk of employee overload and burnout. Organisations must therefore ensure that adequate resources are provided to support employees in meeting these demands (7). This includes providing clear role definitions, access to information, training and development opportunities, and supportive supervision. By aligning demands with available resources, organisations can reduce the likelihood of stress-related outcomes and promote positive employee experiences (10). Importantly, this balance must be monitored continuously, as the demands associated with change processes may evolve over time. Regular assessment of employee experiences can help organisations identify emerging risks and respond proactively.

### 7.6 Implications for policy and organisational practise

The proposed framework has important implications for organisational policy and practise. Organisations should integrate employee wellbeing considerations into their change management policies, ensuring that strategies are aligned with both organisational objectives and employee needs. This may include incorporating wellbeing assessments into change planning processes, monitoring employee wellbeing during implementation, and evaluating the long-term impact of change initiatives. Such approaches enable organisations to identify potential risks early and implement targeted interventions to mitigate negative outcomes (2).

Furthermore, organisations should recognise that employee wellbeing is not only an ethical responsibility but also a strategic priority. Supporting employee wellbeing during organisational change contributes to improved organisational performance, increased engagement, and reduced turnover, thereby enhancing overall organisational effectiveness (10).

## 8 Implications limitations

This study is conceptual in nature and therefore does not provide empirical evidence to test the relationships proposed within the framework. The model has been developed through theoretical integration and synthesis of existing literature and should therefore be viewed as a foundation for future empirical investigation rather than as a confirmed explanation of employee wellbeing outcomes.

In addition, the framework draws primarily from organisational psychology, occupational health, and change management literature. Although the framework is intended to be broadly applicable, organisational, cultural, and sectoral differences may affect the extent to which the proposed relationships operate in practise. Future research is therefore needed to examine the framework across different contexts and employee populations.

## 9 Implications future research

Future research should focus specifically on addressing the conceptual gaps identified in the literature and explored through the proposed framework. In particular, empirical studies are needed to examine the integrated relationships between organisational change, resource loss, job demands, job resources, and contextual organisational resources. Research is also required to determine whether mental health consistently functions as a central dimension of employee wellbeing across different organisational, cultural, and industry contexts. Furthermore, future studies should investigate how leadership, communication, employee involvement, and psychological safety interact collectively to influence employee adaptation during organisational change.

Future research could also explore industry-specific and cultural variations in employee wellbeing outcomes during organisational transformation. Given the increasing prevalence of digital transformation and artificial intelligence in contemporary workplaces, additional research is needed to understand how these developments function as sources of both job demands and job resources. Such studies would contribute to refining and extending the proposed framework while strengthening understanding of employee wellbeing in changing work environments.

## 10 Conclusion

The context of increasing global uncertainty, intensified by the long-term effects of COVID-19, underscores the importance of a human-centred approach to organisational change. In environments where instability, inequality, and resource constraints remain prevalent, decisions about how change is implemented are not only strategic but also ethical. While the framework is designed to be broadly applicable, its emphasis on support, fairness, and psychological safety is particularly critical in contexts where employee vulnerability is heightened and trust in organisational systems may be fragile.

As workplace transformation continues to evolve, the need to integrate mental health considerations into organisational strategies will become increasingly urgent. In response to this challenge, this paper developed an integrative conceptual framework that combines Conservation of Resources theory and the Job Demands-Resources model to explain how organisational change influences employee wellbeing and mental health through the interaction of resource loss, job demands, job resources, and contextual organisational resources. By addressing key gaps in the existing literature and providing a foundation for future empirical investigation, the framework offers a more comprehensive understanding of employee adaptation during organisational change. Ultimately, sustainable organisational transformation depends not only on achieving strategic objectives but also on protecting and strengthening the psychological resources that enable employees to thrive in changing work environments.
